# Sinus Lift and Implant Insertion on 3D-Printed Polymeric Maxillary Models: Ex Vivo Training for In Vivo Surgical Procedures

**DOI:** 10.3390/jcm10204718

**Published:** 2021-10-14

**Authors:** Diana Florina Nica, Alin Gabriel Gabor, Virgil-Florin Duma, Vlad George Tudericiu, Anca Tudor, Cosmin Sinescu

**Affiliations:** 1 School of Dental Medicine, “Victor Babes” University of Medicine and Pharmacy of Timisoara, 2A Eftimie Murgu Place, 300070 Timisoara, Romania; nica.diana@umft.ro; 2 Research Center in Dental Medicine Using Conventional and Alternative Technologies, School of Dental Medicine, “Victor Babes” University of Medicine and Pharmacy of Timisoara, 9 Revolutiei 1989 Ave., 300070 Timisoara, Romania; gabor.alin30@gmail.com (A.G.G.); anca.ancutza@gmail.com (A.T.); minosinescu@gmail.com (C.S.); 3 3OM Optomechatronics Group, Faculty of Engineering, “Aurel Vlaicu” University of Arad, 2 Elena Dragoi, 310177 Arad, Romania; 4 Doctoral School, Polytechnic University of Timisoara, 1 Mihai Viteazu Ave., 300222 Timisoara, Romania; 5 Dentavis Timisoara, 20B Popa Sapca Str., 300057 Timisoara, Romania; vladtudericiu@gmail.com

**Keywords:** dental implants, sinus lift, ex vivo training procedure, in vivo surgery procedure, 3D printing, CAD/CAM techniques, maxillary models, Cone Beam Computed Tomography (CBCT), polymers

## Abstract

Background and Objectives: The aim of this study is to demonstrate the increased efficiency achieved by dental practitioners when carrying out an ex vivo training process on 3D-printed maxillaries before performing in vivo surgery. Materials and Methods: This developed ex vivo procedure comprises the following phases: (i) scanning the area of interest for surgery; (ii) obtaining a 3D virtual model of this area using Cone Beam Computed Tomography (CBCT); (iii) obtaining a 3D-printed model (based on the virtual one), on which (iv) the dental practitioner simulates/rehearses ex vivo (most of) the surgery protocol; (v) assess with a new CBCT the 3D model after simulation. The technical steps of sinus augmentation and implant insertion could be performed on the corresponding 3D-printed hemi-maxillaries prior to the real in vivo surgery. Two study groups were considered, with forty patients divided as follows: Group 1 comprises twenty patients on which the developed simulation and rehearsal procedure was applied; Group 2 is a control one which comprises twenty patients on which similar surgery was performed without this procedure (considered in order to compare operative times without and with rehearsals). Results: Following the ex vivo training/rehearsal, an optimal surgery protocol was developed for each considered case. The results of the surgery on patients were compared with the results obtained after rehearsals on 3D-printed models. The performed quantitative assessment proved that, using the proposed training procedure, the results of the in vivo surgery are not significantly different (*p* = 0.089) with regard to the ex vivo simulation for both the mezio-distal position of the implant and the distance from the ridge margin to sinus window. On the contrary, the operative time of Group 1 was reduced significantly (*p* = 0.001), with an average of 20% with regard to in vivo procedures performed without rehearsals (on the control Group 2). Conclusions: The study demonstrated that the use of 3D-printed models can be beneficial to dental surgeon practitioners, as well as to students who must be trained before performing clinical treatments.

## 1. Introduction

In dental clinics, patients often have terminal edentulism that cannot be treated using traditional prosthetic methods [1,2,3]. Also, sometimes they do not accept a mobile/removable prosthesis. In such situations, to restore the essential functions of the stomatognathic system, implant therapy must be used. Such a technique requires an additional sinus lift surgery [4,5] as well as bone augmentation for the insertion of implants [6,7,8,9]. Despite the drawback represented by the necessity for surgery, by following such procedures, most patients can benefit from an optimal prosthetic restoration from both a morphological and functional point of view.

To optimize the necessary in vivo procedures, extra support can be provided by three-dimensional (3D) printing. The scope of using this technique is to be able to rehearse, ex vivo, the surgical maneuvers before performing them on the patient. Therefore, the expected rate of success in vivo may increase, as well as the precision and time-efficiency of the intervention. In this respect, craniofacial surgery represents one of the top areas where 3D printing technology has built up interest [10,11,12]. Thus, precise individual patient models help practitioners in reconstruction in planning and morphological rehabilitation. Also, they can serve as teaching and educational tools [13,14].

In general, 3D printing, also known as rapid prototyping, is a process of forming a solid object of a required shape. It was first described and patented in 1986 by Charles Hull [15] and in 1992 by Deckard et al. [16]. An additive manufacturing (AM) process, in which successive layers of material are set in different shapes, is used. This process is distinct from traditional (i.e., subtractive) machining techniques, which rely on eliminating materials using methods such as cutting or drilling, starting from a semi-manufactured part. In contrast, 3D printing starts from a digital model. Through AM, the desired object is created layer by layer, until it reaches its final shape, using a range of possible methods [17,18,19]. Efforts to optimize related techniques, especially scanning [20,21,22], have been made to increase parameters of the process such as precision, surface roughness, and size of objects to be printed.

In the present work, maxilla models are obtained through 3D printing using Computer-Aided Design/Computer-Aided Manufacturing (CAD/CAM). The design of the 3D-printed models is based on the clinical data obtained from intraoral scanning, as well as on paraclinical data retrieved from the patients using Cone Beam Computed Tomography (CBCT). The obtained 3D-printed models are utilized for ex vivo training for implant insertion and sinus lift simulations before performing the real, in vivo surgery in the clinical area.

The first hypothesis of the study is to prove that using such implant simulations and clinical procedure rehearsals provides a superior quality of prosthetic restorative treatments on dental implants. To verify this hypothesis, a comparison must be made between the clinical results obtained in the final in vivo procedure and those obtained when the ex vivo training on models is performed.

The second hypothesis is that intervention time is reduced when such simulations are performed, compared to the situation when they are not utilized. To verify this hypothesis, a comparison must be made between the operative time necessary for the final in vivo procedure when such simulations (and the corresponding ex vivo training on models) are performed and the operative time necessary without this developed procedure. 

## 2. Materials and Methods 

Forty patients were included in this study. They were divided into two groups: 

Group 1 comprised twenty patients selected for the developed clinical procedure, which included simulations/rehearsals, as described in the following for one of these clinical cases. Similar procedures were performed on each of these twenty patients. 

Group 2 was a control group, consisting of other twenty treated patients on which in vivo surgery was performed without the training developed in this work. This group was considered in order to evaluate the second hypothesis of this study. As a necessary remark, the cases of Group 2 were selected from a larger group of patients on which the type of surgery specific to this study was performed previously to developing the simulation and rehearsal procedure developed in this work. The selection was made by considering an as good as possible match (in pairs of patients) with the cases of Group 1 from the point of view of their clinical difficulty (in this respect, please see Section 3.2). 

This work was approved by the Committee of Medical Research of the “Victor Babes” University of Medicine and Pharmacy of Timisoara, Romania, following the Ethical protocol of the University with the CECS Approval no. 09/02.03.2018, and it was carried out according to guidelines of the Declaration of Helsinki. Informed consent was submitted to all of the forty enrolled patients.

### 2.1. Diagnosis

As mentioned above, a clinical case selected from Group 1 is presented in the following to describe the method, i.e., the developed simulation and rehearsal procedure. 

**Figure 1:**
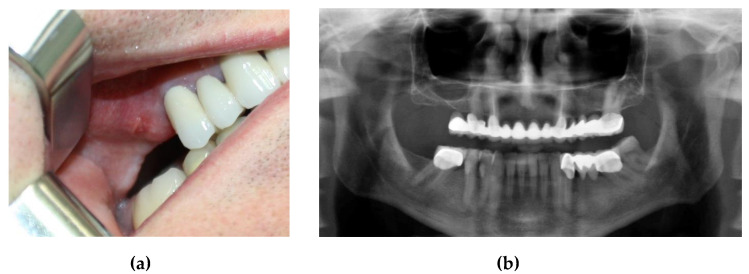
(**a**) Initial clinical assessment; (**b**) preoperative orthopantomogram (OPG) showing an insufficient height for implant insertion in the maxillary right first molar edentulous site.

This case involved a 50-year-old male patient who presented to the clinic with a maxillary cantilever cemented prosthetic restoration. The patient affirmed that the functional role of this restoration became completely inadequate, with no possibility of mastication on the right side. The first step of the procedure included a clinical examination (Figure 1), where the missing maxillary right first and second molars were remarked. The first molar was extracted two years ago and the second molar over ten years ago. The intraoral examination showed a healthy gingival and mucosal status. For an accurate diagnosis, a radiological evaluation was performed. The treatment options were explained, and the patient was also informed about the benefits and risks of each treatment option. As he wanted to avoid a removable prosthesis due to its inconvenience, the chosen treatment option was an implant-supported crown restoration.

**Figure 2:**
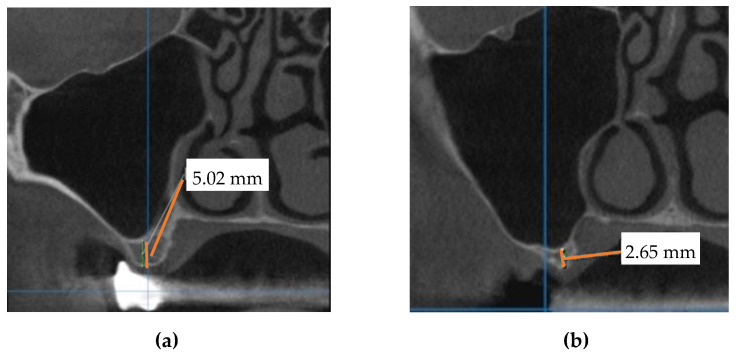
Cross-sectional CBCT views corresponding to: (**a**) the 16 with a bone height of 5.02 mm; (**b**) the 17 with a bone height of 2.65 mm and insufficient crestal width.

In this first step, a CBCT scan of the maxilla was performed to evaluate the bone anatomy and confirm the absence of the maxillary sinus pathology (Figure 2).

### 2.2. Manufacturing of Hemi-Maxillary 3D Printed Model

**Figure 3:**
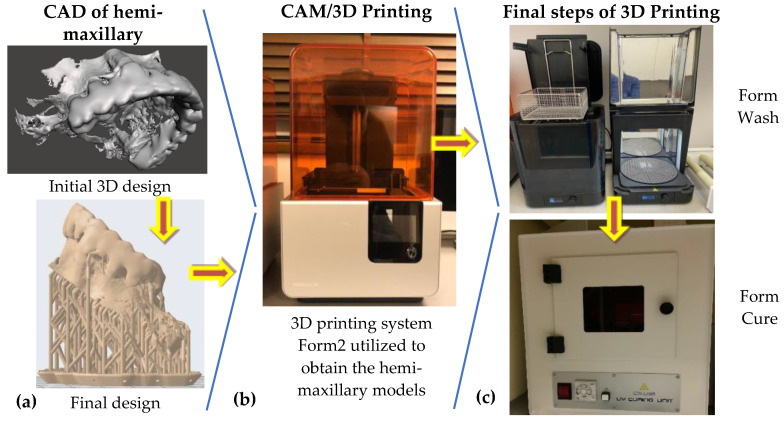
The steps of the design and manufacturing of a hemi-maxillary: (**a**) CAD-initial and final design; (**b**) CAM-3D printing, with a Form2 system (Formlabs Inc., Somerville, MA, USA); (**c**) final two steps of the manufacturing process, i.e., washing and curing of the obtained polymer-based 3D-printed models.

An overview of *this second step of the procedure* is made in Figure 3. First, a digital intraoral impression was performed using the digital intraoral scanning system Cerec OMNICAM (Dentsply Sirona CEE Central, Bucharest, Romania). Thus, an initial 3D design was obtained, as shown in the upper image in Figure 3a. Subsequently, the patient was sent to the radiology center where a CBCT type scan was performed using CRANEX 3DX (Soredex, Tuusula, Finland). A radiographic CBCT scan allows getting an optimally printed pattern due to its in-depth penetration of the tissues, as shown in the lower image in Figure 3a.

The obtained data were overlaid to reach the optimal design of the future 3D-printed model. With the help of this design, a polymeric model was obtained using the 3D printing system Form2 (Formlabs Inc., Somerville, MA, USA). The initial 3D design of the maxillary ready for printing (upper image of Figure 3a) was further correlated with the parameters of the printing system. The printing process of the final 3D design (bottom of Figure 3a) was carried out with the following system characteristics and parameters (Figure 3b): 3D printing can be carried out in slices, with thicknesses ranging from 0.01 to 0.10 mm at a temperature of 22 to 25 °C and a relative humidity of 60%. The 3D printing time of each model was around 6 h. After this process, the scaffold sites were washed and then introduced for 30 min in an oven curing from the same 3D printing system (Figure 3c). The obtained parts were finally processed to remove small excesses of material.

**Figure 4:**
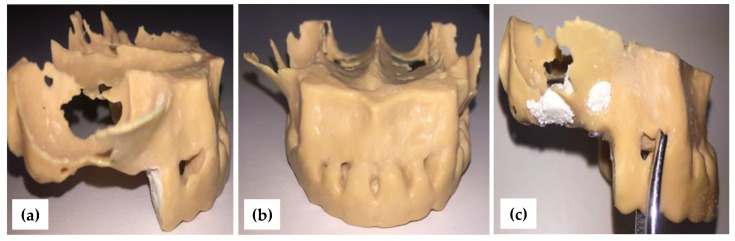
(**a**) 3D-printed model, with (**b**) frontal and (**c**) lateral view.

The Digital 3D Printer Form 2 in Figure 3c, which uses laminated light-curing resins (DigitalWax^®^ DC-Series Casting Resin, DWS, Thiene, Italy), was employed to obtain the 3D polymeric model of the hemi-maxilla for each considered case/patient. Figure 4 shows the 3D-printed polymeric-made hemi-maxilla model of this patient, in different views.

### 2.3. Ex Vivo Simulation of Surgical Procedure

In this third step of the procedure, the dental practitioner performed on the polymeric 3D-printed hemi-maxilla model the simulation of the sinus lift procedure by lateral approach, the insertion of the ex-vivo implant, as well as the later and crestal augmentation. Thus, the surgeon was able to observe the exact positioning of the bone formation, the thickness of the remaining bone, and the distance to the sinus membrane. As a necessary remark, the following operative phases described in the following section could not be rehearsed on the 3D-printed model: incision, flap elevation, sinus membrane elevation, flap repositioning, and suture.

### 2.4. Surgical Procedure

The real operation was performed between 2 to 5 days (maximum) after the rehearsal, depending on the availability of the operation room and the surgeon. The essential criterium was to have the same healthy sinus condition for the patient.

**Figure 5:**
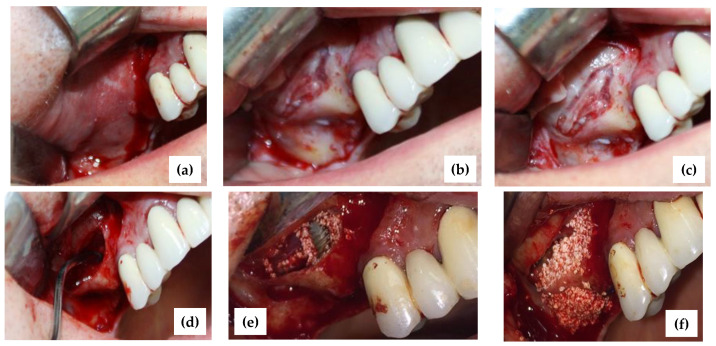
(**a**) Flap design; (**b**) the bony window; (**c**) the bony window, almost completed saving the blood vessels; (**d**) the Schneiderian membrane was elevated; (**e**) inserted implant in the 16th position with simultaneous sinus augmentation; (**f**) sinus lift procedure 17 and lateral crest augmentation.

In this fourth step of the procedure, correlating clinical information with preclinical data, as well as with those obtained on 3D-printed models, the medical doctor was able to pass to the surgical procedure for the sinus lift and the insertion of dental implants, as shown in the example in Figure 5 for the considered patient.

The first clinical stage was the sinus lift. For this case, a sinus floor augmentation procedure via the lateral window approach with simultaneous implant placement for the missing 16 and a sinus augmentation for latter implantation for 17 were planned. On the day of the surgery, this patient received 2 g of amoxicillin one hour before the procedure as antibiotic prophylaxis: 1 g one hour before the procedure and the rest of the dose 12 h after the first dose. As post-operative therapy, the patient received 1 g three times a day per os for 7 days [23]. Following the administration of local anesthesia with articaine, a crestal incision was performed, as well as two vertical releasing incisions on the distal aspect of the maxillary right second premolar and the mesial aspect of the edentulous crest of the third molar, respectively. The incisions were made with a 15C scalpel blade and a full-thickness mucoperiosteal flap was reflected with a lateral sinus wall exposure (Figure 5a).

A bony window was then traced in the 16 and 17 regions using a piezo-surgical unit (Figure 5b,c). The initial bone marking was done using tip SL1. This was followed by the deepening of the mark using SL2. Using the SL3 tip, the Schneiderian membrane on the windows edges was easily elevated (Figure 5d). Following the complete membrane elevation extending to the anterior and medial walls, the implant osteotomy was performed. 

A C1 Implant 3.75 mm × 11.5 mm Standard Platform Conical Connection (MIS Implants Technologies Ltd., Fair Lawn, NJ, USA) with simultaneous sinus lift procedure was inserted on the 16 position (Figure 5e). A dedicated xenograft was compacted into the sinus cavity (CompactBone^®^ B., Dentegris GmbH, Monheim am Rhein, Germany). This specific augmentative material was chosen because of its mineral composition, physical, chemical, and biological properties, as well as because of its hydrophilic surface, with a close resemblance to the characteristics of the human bone. Also, this material allows for fast revascularization because of its interconnecting pore system. Thus, it was able to provide long-term stability of the augmented site with predictable results [24,25].

At the level of 17, a sinus elevation procedure with a simultaneous lateral and vertical augmentation of the crest was performed (Figure 5f). The augmented area was completely covered with a collagen membrane (BoneProtect^®^ Membrane, Dentegris GmbH, Monheim am Rhein, Germany). 

The incisions were closed with interrupted sutures 4-0 polyester (Resorba Medical GmbH, Nürnberg, Germany).

### 2.5. Assessment of the Surgical Procedure

A CBCT was performed the day after the surgery in the fifth step of the procedure. Thus, each patient was followed up two weeks after the surgery to check if the healing was uneventful. Also, no complications occurred in the six-month period during the osteointegration of implants.

**Figure 6:**
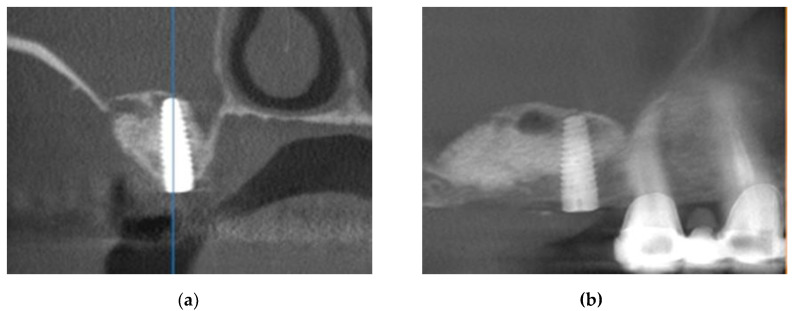
(**a**) Postoperative control of the patient using 3D imaging; (**b**) the augmented 17 site (cross-sections).

For the considered case, this CBCT assessment is presented in the cross-sections in Figure 6. Measurements were then performed, as presented in the following section.

All the phases above were performed, for each patient in the study, by the same surgeon. All the CBCT examinations were performed by the same radiologist.

## 3. Results

### 3.1. Positioning Assessments

**Figure 7:**
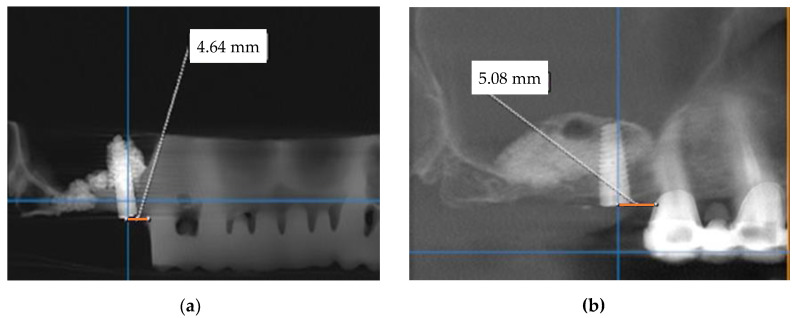
Measurement of the mezio-distal position of the implant (i.e., distance from the anterior tooth to the middle of the implant (**D1**)) on (**a**) the training CBCT model (**D1-3D**) and on (**b**) the postoperative CBCT (**D1-P**)—panoramic view.

**Figure 8:**
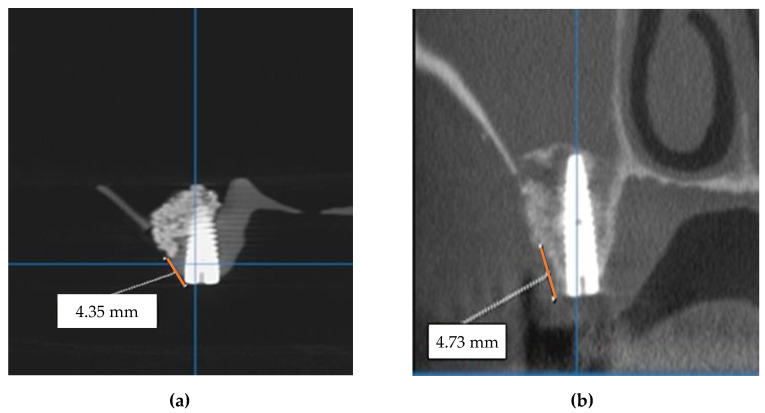
Measurement of the distance between the top of the ridge and the lowest access cut at the buccal wall of the maxillary sinus (for patient number 6 in Table 1) on (**a**) the training CBCT model (**D2-3D**) and on (**b**) the postoperative CBCT (**D2-P**) cross-section.

Following the described procedure, a comparison was made between the mezio-distal positions of the implant on the maxillary arch on the CBCT of the model and the patient’s postoperative CBCT, as shown in the panoramic views in Figure 7. Also, the distances from the top of the maxillary ridge to the lowest cut on the maxillary sinus buccal wall were measured on the cross-section in Figure 8.

Table 1 provides the results of the measurements performed on the CBCT made on the 3D-printed model after the implant insertion versus the CBCT made on the patients after the final (i.e., real clinical procedure of) implant insertion.

The statistical processing of these results was performed using SPSSv17, calculating the mean and the standard deviations (SD). Descriptive statistics were performed for the numerical variables and comparisons between these series of pair values (i.e., **D1-3D** and **D1-P**) were done with the non-parametric Wilcoxon Signed Ranks test (Table 2). The results were considered significant for a value of *p* < 0.05 (Table 3). As it may be concluded from these data, the difference between the distances from the anterior tooth to the middle of the implant in the two considered situations is statistically insignificant (Wilcoxon Signed Ranks Test, *p* = 0.089).

Similar statistical processing was performed using SPSSv17 for the distance from the ridge margin to sinus window, in a comparative look between the data obtained when rehearsing on 3D-printed models (**D2-3D**) and when performing implant procedures on patients (**D2-P**). Descriptive statistics were performed for the numerical variables. Comparisons between these series of pair values were done with the non-parametric Wilcoxon Signed Ranks test (Table 4). The results were considered significant for a value of *p* < 0.05 (Table 5). The difference between these distances was also statistically insignificant (Wilcoxon Signed Ranks Test, *p* = 0.350).

### 3.2. Operative Time

Another important aspect of a surgical procedure, essential from the patient’s point of view, is the operative time. We registered the corresponding operative time intervals T for the procedures described above for the patients in Group 1 (i.e., with performed rehearsals on 3D-printed models) and compared them to the time intervals **T_0_** necessary for the same surgery procedures for the patients in Group 2 (i.e., for the twenty clinical cases that did not benefit from a simulation/rehearsal prior to the surgery). The comparison between these time intervals for each of the twenty patients considered in the control Group 1 and in the study Group 2 was made in Table 6.

The twenty clinical cases of the control Group 2 (numbered 1_0_ to 20_0_ in Table 6) were selected to be an as good as possible match (from the point of view of the clinical difficulty) with the twenty cases selected for the study Group 1. This relative match of the cases presented in parallel in Table 6 for Groups 1 and 2 is an inherent limitation of the present work, as one cannot use the same clinical cases for both analyzes. Instead, cases with similar levels of difficulty from a clinical point of view must be selected for an as rigorous as possible comparison.

Statistical processing was performed using SPSSv17 for these operative times, similar to Section 3.1, in the comparative look between the necessary time without rehearsal previous to the procedure (**T_0_**), i.e., for Group 2, and when implant procedures on patients were performed after such a rehearsal on 3D-printed models (**T**), i.e., for Group 1. Descriptive statistics were performed for the numerical variables and comparisons between these series of pair values using the non-parametric Wilcoxon Signed Ranks test (Table 7). 

The mean time **T_0 mean_** required for implant placement with a simultaneous sinus lift procedure in patients of Group 2 (i.e., without rehearsals) was 117.55 min. The mean time **T_mean_** needed for the same procedure in the cases of the patients of Group 1 (i.e., with rehearsals on 3D-models prior to the real surgery) was 93.2 min. The 24.35 min difference between these two values, as well as the relative decrease (**T_0 mean_** − **T_mean_**)/**T_0 mean_** equal to 20.71% from the **T_0_** surgery time indicates the benefit of applying the rehearsal procedure for patients.

The results were considered significant for a value of *p* < 0.05 (Table 8). The difference between these distances was statistically significant (Wilcoxon Signed Ranks Test, *p* < 0.001).

## 4. Discussion and Conclusions

Surgeons often use different 3D-printed human organs before surgery, for example in craniofacial reconstruction [26,27] and implant-site assessment [28]. Thus, they can provide a more detailed picture of certain anatomical aspects than when using classical two-dimensional (2D) models or 3D computer imagery. Using 3D-printed parts, surgeons can physically feel the replicas of those organs, bone structures, or other aspects of interest. Thus, they can perform the surgery protocol as a training procedure. An alternative is to perform implant surgical training and research on a cadaver specimen.

With 3D printing, dental medicine can also evolve in terms of the quality of future implant surgical treatments, and this has been approached in numerous studies [12,29,30,31,32,33,34]. Materials and manufacturing procedures have been explored [35,36], as well as safety and efficacy issues [37]. Accuracy evaluations of 3D-printed models for mandibular models were also performed, using a range of methods [38].

On the other hand, oral implantology is not a stand-alone branch; it depends equally on the future prosthetic treatment that must be consistent with the insertion of implants [39]. Tissue engineering is another aspect to be considered, both for bone [7,8,9,40,41,42] and soft tissue [43]. Clearly, in oral implantology, various clinical situations may arise that require optimal thinking and the calculation of the treatment plan.

The present study approached the utility of the 3D-printed models in the visualization of the complex maxillary anatomical structures and the impact of the surgery simulation before the in vivo operative time. Both maxillary implant insertion combined with sinus augmentation procedures require an accurate technique. Guidance is necessary to achieve the appropriate angulation and position in relation to the adjacent teeth, as well as a preplanned design and positioning of the lateral window for approaching the sinus. 

Due to the realistic form of the 3D-printed models, the technical steps of sinus augmentation and implant insertion could be performed on the corresponding model prior to surgery. Technical steps of the procedure were rehearsed in the simulation using the 3D model (as detailed in the Materials and Methods section): shape and size of antrostomy, implant insertion, and grafting. As pointed out, several operative phases could not be rehearsed on the 3D-printed model: incision, flap elevation, sinus membrane elevation, flap repositioning, and suture (Figure 5).

In this work, we analyzed the compliance of the ideal mezio-distal position of the implant due to its crucial role for the final prosthetic restoration and the health of the tissue around it [28]. The statistical data in Table 2, Table 3, Table 4 and Table 5 demonstrated that no significant differences were obtained between simulations and clinical procedures. Thus, the preplanned positioning of the implant on the 3D-printed model was transferred in the in vivo operative time. The design and position of the lateral window during the sinus lift procedure have significant roles in avoiding intra- and post-surgical complications [44,45]. This is the reason why we have chosen to analyze the compliance of the ideal position of the lateral window. This method allowed us to assess the thickness of the lateral maxillary wall and its convexity from the practical scene. From the considered clinical cases, in four of them, the buccofacial dimensions of the lateral maxillary wall were thicker than 4.5 mm. In these specific cases, the 3D models allowed to undergo the osteotomies and to practice this challenging mandatory step of sinus augmentation. The thick lateral maxillary wall changed the standard surgical procedure, meaning the necessity of thickness reduction of the trap-door window, a time-consuming maneuver. In the other three cases, we found a convexity of the lateral maxillary sinus wall. The 3D models again proved useful, as they allowed to evaluate the surgical access to the sinus by considering the high risk of Schneiderian membrane perforation. 

As an overall result, the statistical data showed no significant differences between the 3D-printed model and postoperative CBCTs.

The first hypothesis of the study was proved: using implant simulations allows for a superior quality of future prosthetic restorative treatments on dental implants. The second hypothesis was also confirmed: a significant reduction of the intervention time of around 20% was obtained (Table 7 and Table 8) when rehearsals on 3D-printed models were performed, compared to the situation when they were not utilized.

In the complex clinical cases approached, the 3D-printed models allowed for a precise plan of the surgery. Also, the transfer of the accuracy in the real operation field was made possible. Therefore, using 3D-printed models, implants can be inserted in vivo in full compliance with future prosthetic treatment. Improving the surgical operative time, as obtained in this study, is a real benefit to both the patient and the surgeon. An accurate sinus lift procedure along with reduced tissue manipulation leads to a better perception during the recovery time.

In conclusion, the use of 3D-printed models can benefit implant practitioners, as well as students taking a course in Oral Implantology. Also, it can be a support to students of dental medicine in implantology to simulate implant insertions. Thus, 3D-printed models can play a significant role in the educational field. The anatomical models mitigate the inconvenience of cadaveric dissection, allowing the student to focus only on the structural anatomy and to relate it with its functional role [45]. In contrast, commercial anatomical models, compared with the 3D models, are only illustrative and unable to reproduce the biological particularities [46]. For a more pragmatic model used for a hands-on simulation, creating a double-material 3D model could be more appropriate. In the cases considered in our work, this would imply simulating the solid bone and the elastic Schneiderian membrane. However, a printer that could create models that are able to match the properties of the tissues involved in the operation represents a major issue, because the current 3D printing technologies do not incorporate fluids into their 3D-printed objects. Printing with dissectible hydro-gel materials could get us closer to models which could be felt and could deform similar to living tissues [47].

We expect that further progress in the field will lead to the development of both soft and hard printable materials that mimic the haptics of biological tissues. The cost-effective availability of 3D printers may allow practitioners to enhance their surgical skills on 3D-printed models, with a resolution similar to one of the original dissectible structures. 

For the imaging part, future work in this direction in our group includes using combined Optical Coherence Tomography (OCT) [48,49,50] and 3D CBCT [51] for the assessment of the results of such clinical procedures, as we have demonstrated both the complementarity [52] and the optimization [53] of the latter imaging technique by using the former.

## Figures and Tables

**Table 1:** Measurements performed on CBCT images: 3D-printed models (rehearsed procedures) versus patients (postoperative).

Patient	CBCT Measurements on 3D-Printed Models (mm)		CBCT Measurements on Postoperative Patients (mm)	
	Distance from the Anterior Tooth to the Middle of the Implant (D1-3D)	Distance from the Ridge Margin to the Sinus Window (D2-3D)	Distance from the Anterior Tooth to the Middle of the Implant (D1-P)	Distance from the Ridge Margin to the Sinus Window (D2-P)
1	4.23	4.47	4.25	4.72
2	3.91	5.10	4.01	4.87
3	4.43	4.87	4.47	4.64
4	3.86	4.58	3.92	4.98
5	4.67	5.75	4.51	5.68
6	4.64	4.35	4.78	4.73
7	3.80	4.29	3.82	4.48
8	3.86	5.67	3.91	5.39
9	3.90	4.92	4.17	5.19
10	3.92	5.17	4.03	4.83
11	4.45	4.77	4.27	4.91
12	4.29	4.57	4.37	4.31
13	3.95	4.86	3.94	5.07
14	4.17	5.14	4.26	4.92
15	3.83	5.12	4.15	5.37
16	3.89	4.93	4.06	5.19
17	4.57	4.62	4.41	4.95
18	4.49	4.71	4.45	4.58
19	4.54	5.12	4.52	5.34
20	4.48	4.72	4.51	4.60

**Table 2:** Descriptive statistics regarding the distance from the anterior tooth to the middle of the implant following rehearsal procedures on 3D-printed models (**D1-3D**) compared to the distance from the anterior tooth to the middle of the implant following implant procedures on patients (**D1-P**).

Distance	N	Mean (mm)	SD (mm)	Minimum (mm)	Maximum (mm)
**D1-3D**	20	4.194	0.316	3.80	4.67
**D1-P**	20	4.241	0.260	3.82	4.78

**Table 3:** Ranks regarding the **D1-3D** distances compared with the **D1-P** distances (the Wilcoxon Signed Ranks test results).

		N	Mean Rank (mm)	Sum of Ranks (mm)
**D1-P** versus **D1-3D**	Negative Ranks	6 ^a^	9.92	59.50
	Positive Ranks	14 ^b^	10.75	150.50
	Ties	0 ^c^	-	-
	Total	20	-	-

Remarks: ^a^ **D1-P** < **D1-3D**; ^b^ **D1-P** > **D1-3D**; ^c^ **D1-P** = **D1-3D**.

**Table 4:** Descriptive statistics regarding the distance from the ridge margin to the sinus window for rehearsals on 3D-printed models (**D2-3D**) and the distance from the ridge margin to the sinus window for implant procedures on patients (**D2-P**).

Distance	N	Mean (mm)	SD (mm)	Minimum (mm)	Maximum (mm)
**D2-3D**	20	4.887	0.385	4.29	5.75
**D2-P**	20	4.936	0.347	4.31	5.68

**Table 5:** Ranks regarding the **D2-3D** distances compared to the **D2-P** distances (the Wilcoxon Signed Ranks test results).

		N	Mean Rank	Sum of Ranks
**D2-P** versus **D2-3D**	Negative Ranks	9 ^a^	8.89	80.00
	Positive Ranks	11 ^b^	11.82	130.00
	Ties	0 ^c^	-	-
	Total	20	-	-

Remarks: ^a^ **D2-P** < **D2-3D**; ^b^ **D2-P** > **D2-3D**; ^c^ **D2-P** = **D2-3D**.

**Table 6:** Operative time intervals of implant procedures without (**T_0_**) versus with 3D-model-based simulations/rehearsals (**T**).

Patient of (Control) Group 2	Operative time Without 3D-Model-Based Rehearsal T_0_ (min)	Patient of Study Group 1	Operative Time with Performed Simulation/Rehearsal T (min)
1_0_	128	1	89
2_0_	101	2	92
3_0_	134	3	102
4_0_	112	4	88
5_0_	115	5	102
6_0_	142	6	79
7_0_	123	7	82
8_0_	126	8	98
9_0_	98	9	95
10_0_	105	10	100
11_0_	141	11	87
12_0_	105	12	81
13_0_	113	13	78
14_0_	124	14	97
15_0_	99	15	103
16_0_	133	16	91
17_0_	90	17	101
18_0_	131	18	120
19_0_	102	19	85
20_0_	129	20	94

**Table 7:** Descriptive statistics regarding the operative time without rehearsals (**T_0_**) and with performed simulations/rehearsals on 3D-printed models (**T**).

Time	N	Mean (min)	SD (min)	Minimum (min)	Maximum (min)
**T_0_**	20	117.55	15.55	90	142
**T**	20	93.2	10.21	78	120

**Table 8:** Ranks regarding the **T_0_** and the **T** operative times.

		N	Mean Rank	Sum of Ranks
**T_0_** versus **T**	Negative Ranks	18 ^a^	11.25	202.50
	Positive Ranks	2 ^b^	3.75	7.50
	Ties	0 ^c^		
	Total	20		

Remarks: ^a^ **T** < **T_0_**; ^b^ **T** > **T_0_**; ^c^ **T** = **T_0_**.

## Data Availability

Data are available on request from D.F.N.

## Abbreviations

Three dimensional	3D
Cone Beam Computed Tomography	CBCT
Computer-Aided Design	CAD
Computer-Aided Manufacturing	CAM
Additive manufacturing	AM
Distance from the anterior tooth to the middle of the implant	**D1-3D**
Distance from the ridge margin to the sinus window	**D2-3D**
Distance from the anterior tooth to the middle of the implant	**D1-P**
Distance from the ridge margin to the sinus window	**D2-P**
Standard deviation	(SD)
Operative time without 3D-model-based rehearsal	**T_0_**
Operative time with prior-performed rehearsal	**T**
Optical Coherence Tomography	OCT
